# miR-129 Attenuates Myocardial Ischemia Reperfusion Injury by Regulating the Expression of PTEN in Rats

**DOI:** 10.1155/2021/5535788

**Published:** 2021-08-14

**Authors:** Zhao-Hui Dai, Zhi-Ming Jiang, Hua Tu, Li Mao, Gui-Lin Song, Zhong-Bao Yang, Fang Liu, Md Sayed Ali Sheikh

**Affiliations:** ^1^The Affiliated Changsha Hospital of Hunan Normal University, Changsha, Hunan 410006, China; ^2^Chest Pain Center of Changsha, Changsha, China; ^3^Department of Basic Medicine, Changsha Health Vocational College, Changsha, Hunan 410600, China; ^4^Institute of Emergency and Critical Care Medicine of Changsha, Changsha, China; ^5^College of Medicine, Hunan Normal University, Changsha, Hunan 410006, China; ^6^Internal Medicine Department, Cardiology, College of Medicine, Jouf University, Sakaka, Aljouf, Saudi Arabia

## Abstract

PTEN/AKT signaling plays pivotal role in myocardial ischemia reperfusion injury (MIRI), and miRNAs are involved in the regulation of AKT signaling. This study was designed to investigate the interaction between miR-129 and PTEN in MIRI. A MIRI rat model and a hypoxia reoxygenation (H/R) H9C2 cell model were constructed to simulate myocardial infarction clinically. TTC staining, creatine kinase (CK) activity, TUNEL/Hoechst double staining, Hoechst staining and flow cytometer were used for evaluating myocardial infarction or cell apoptosis. miR-129 mimic transfection experiment and luciferase reporter gene assay were conducted for investigating the function of miR-129 and the interaction between miR-129 and PTEN, respectively. Real-time PCR and western blotting were performed to analyze the gene expression. Compared to the control, MIRI rats presented obvious myocardial infarction, higher CK activity, increased expression of caspase-3 and PTEN, decreased expression of miR-129, and insufficient AKT phosphorylation. Consistently, H/R significantly increased the apoptosis of H9C2 cells, concomitant with the downregulation of miR-129, upregulation of PTEN and caspase-3, and insufficient phosphorylation of AKT, while miR-129 mimic obviously inhibited the expression of PTEN and caspase-3, increased the AKT phosphorylation, and decreased the cell apoptosis. Additionally, miR-129 mimic obviously decreased the relative luciferase activity in H9C2 cells. To our best knowledge, this study firstly found that the low expression of miR-129 accelerates the myocardial cell apoptosis by directly targeting 3′UTR of PTEN. miR-129 is an important biomarker for MIRI, as well as a potential therapy target.

## 1. Introduction

myocardial infarction is one of the leading causes of death worldwide, especially in elder population, which was characterized by the blockage of blood to coronary artery and consequently resulting in dysfunction of myocardial cell, even death [1]. Restoration of blood to the coronary artery by pharmacological or surgery approaches was the common and effective therapy clinically. However, besides the benefits to the affected heart, blood reperfusion will paradoxically be harmful to the heart at certain stage, which was called myocardial ischemia/reperfusion injury (MIRI) [2]. The common and worst consequence of MIRI is myocardial cell death that was associated with the inhibition of antiapoptotic signaling pathways [3]. Among them, Akt signaling pathways are involved in many cellular physiological processes and have important roles in cell survival [4]. Amounted evidences demonstrated that MIRI contributes to the decrease of phosphorylation of AKT in myocardial cell [4, 5]. Thereby, elucidation of the molecular mechanism of decreased AKT phosphorylation in MIRI is very important for the knowing of myocardial infarction as well as its therapy. As an upstream molecule of the AKT signaling pathway, PTEN plays a pivotal role in AKT phosphorylation regulation. Previous studies have found that higher expression levels of PTEN were, at least partially, the cause of insufficiency of AKT phosphorylation in myocardial cells in pathological state caused by MIRI [6]. Although there are many experiments designed to study the expression regulation mechanism of PTEN in MIRI and got many findings, its detail mechanism still needs to be elucidated.

miRNAs are a class of conserved noncoding RNAs of about 22 nt in length and have many biological functions; for example, it can suppress gene expression [7]. Previous studies have proved that one miRNA can target several different genes and one gene can be targeted by several different miRNAs. [7] For its broad biological functions, miRNAs play key roles in the development and progress of diseases, such as myocardial infarction and tumor. [8, 9] Accumulated evidence show that abnormal expression of miRNAs was associated with the process of MIRI, for example, miR-494, miR-26a-5p, and miR-199a [10–12]. In addition, Xing et al. have demonstrated that PTEN is a target gene of miR-26a-5P [10]. This suggested that miRNAs are involved in MIRI, at least partly, by regulating of PTEN. However, few of the present studies were designed to explore the role of the miRNAs/PTEN axis in MIRI. For better understanding the role of miRNA in PTEN expression regulation in MIRI, more target miRNAs of PTEN need to be confirmed. miR-129 was proved to be a multifunction molecule and involving in the development and progression of many diseases, such as cerebral ischemia reperfusion injury and MIRI [13–15]. We recently found that miR-129 is a putative target miRNA of PTEN based on bioinformation analysis and prediction. Thus, explicating the interrelationship between miR-129 and PTEN is of great importance in understanding MIRI.

This study was designed to investigate the role of miR-129 in MIRI. To our best knowledge, this was the first time to observe the interaction of miR-129 and PTEN and it will provide a novel target for MIRI therapy.

## 2. Materials and Methods

### 2.1. Animals' Experiments

A total of 24 male Sprague-Dawley rats (8 weeks old, 250-260 g) were purchased from the Hunan SJA Laboratory Animal Co., Ltd. Animals were housed in conditions (24°C, 60% humidity, and 12 h light/dark cycle) with free access to water and food. The rats were randomly divided into three groups (the control group, sham group, and I/R injury group; 8 per group). A rat MIRI model was established following a previously described surgical method [10]. Firstly, the rats were intraperitoneally injected with 3% pentobarbital sodium (40 mg/kg) for light anesthesia. Then, the hair of the neck was removed and an endotracheal intubation was performed. After that, a rodent ventilator (R407, RWD Life Science, China) and an electrocardiograph (Beheart R3, Mindray, China) were used for monitoring the rats' respiratory rate and electrocardiogram, respectively. Before carrying out left lateral thoracotomy and exposing the heart, the skin along the left edge of the sternum was incised and the muscle between the third and fourth ribs was separated. An 8–0 atraumatic suture was used to ligate the left anterior descending coronary artery (LADCA) to produce occlusions. When the ST segment in ECG was notably upgraded indicated that the coronary artery was successfully blocked. After 30 min of occlusion, the suture was released and the blood to the heart was restored. During the reperfusion process, there is an obvious decline in the ST segment. Thus, the myocardial I/R model was successfully constructed. Next, the pneumothorax was evacuated manually, and the chest and skin were closed using a 6–0 Prolene suture. For analgesia, buprenorphine (0.1 mg/kg body weight) was administered ip. Additionally, the sham group of rats subjected to the same procedure except the ligation of LADCA.

### 2.2. Measurement of Creatine Kinase (CK) Activity

A CK activity test kit (Beijing Solarbio Science & Technology Co., Ltd.) was used for CK activity measurement. According to the manufacture's instruction, 0.1 g of heart tissues and 1 mL extraction solution were mixed to prepare homogenate at 0°C. Then, the homogenate was centrifuged at 10000 g, 4°C for 15 min and the supernatant was collected. The CK activity was determined using a spectrophotometer (colorimetric method). Briefly, a 1000 *μ*L reaction mix was prepared by mixing 200 *μ*L supernatant, 450 *μ*L working solution, and 350 *μ*L water. Then, the absorption value was read at 340 nm. The CK activity was calculated as the following formula: CK (U/g) = 268 × Δ*A* ÷ *W*. (Δ*A* = (the absorption value of sample at the time of 190 s − the absorption value of sample at the time of 10s) − (the absorption value of control at the time of 190 s − the absorption value of control at the time of 10s)).

### 2.3. Determination of Infarction Area

At the end of 2 h reperfusion, all rats (16 rats) were sacrificed by decapitation after loss of consciousness following anesthesia via intraperitoneal injection of 3% pentobarbital sodium (40 mg/kg weight). The heart was immediately excised and stored at –20°C. Prior to TTC staining, heart slices were prepared by coronally cutting heart tissues into sections with a thickness of 0.2-0.3 cm. Then, the slices were immersed in 2% TTC and maintained in dark (37°C) for 0.5 h. The sample pictures were captured. The infarct area was assessed using ImageJ software (version 1.4; National Institutes of Health). The myocardial infarct size is reported as the infarct area.

### 2.4. Cell Culture

The H9C2 cell line was purchased from the Committee on Type Culture Collection of Chinese Academy of Sciences of Shanghai and maintained in a cell incubator with conditions as 37°C, 5% CO_2_, and 95% air. DMEM culture (Thermo Fisher Scientific, Inc.) supplemented with 10% FBS and penicillin/streptomycin (100 U/mL) was used for cell culture. Cells (5 × 10^5^) were cultured with 12-well plates for miRNA functional assays and the luciferase reporter gene experiment.

### 2.5. Cell Model of Hypoxia/Reoxygenation (H/R)

An in vitro H9C2 cell H/R model was established following the methods of Mao et al. to mimic myocardial ischemia reperfusion injury [16]. When cells grow to a 70% area of a plate, the DMEM culture was removed and cells were washed twice using PBS to remove the residual culture. Following that, the cells were incubated with Dulbecco's phosphate-buffered saline (DPBS; Sigma-Aldrich; Merck KGaA) at 37°C in a hypoxic condition (95% N_2_ and 5% CO_2_) for 5 h. Then, the DPBS was dumped and replaced with an addition of DMEM culture, and the cells were maintained at 37°C in a standard condition (5% CO_2_ and 95% air) for 20 h reoxygenation. The attempts of hypoxia/reoxygenation (5 h/20 h) were to get an apoptosis rate of H9C2 cells greater than 40%.

### 2.6. Bioinformatics Prediction

TargetScan (http://www.targetscan.org/vert-72/) and miRBase (http://www.mirbase.org/) were used to predict the target miRNAs of PTEN.

### 2.7. Cell Transfection

miR-129-5p mimic and negative controls (NC) miRNA were synthesized from Shanghai Gene Pharma Co., Ltd. (China). To investigate the function of miR-129 in a cardiomyocyte, H9C2 cells were transfected with miR-129 mimics (final concentration100 nm) by Lipofectamine 2000 (Ruibo, China).

### 2.8. Luciferase Reporter Assay

Luciferase reporter assay was performed following the methods of Mao et al. to observe the interaction between PTEN and miR-129 [16]. A luciferase reporter gene plasmid (pGL6; Promega Corporation) was constructed and designated as PTEN-WT or PTEN-MU according to the sequence of 3′UTR of PTEN cloned into the plasmid whether it contains the wild-type (WT) binding sites of miR-129-5p“CAAAAAA” or mutant (MUT)“CAAGAAA”. These plasmids were verified by electrophoresis (figure S1). The plasmids and miR-129-5p mimic were cotransfected into the H9C2 cell via Lipofectamine® 2000 (Ruibo, China). After 24 h of transfection, the relative luciferase activity of H9C2 cells was determined using a dual luciferase reporter gene assay kit (Beyotime Institute of Biotechnology). Firefly luciferase activities were normalized to Renilla luciferase activities.

### 2.9. TUNEL/Hoechst Double-Labeling

TUNEL/Hoechst double-labeling was performed following the methods of Mao et al. to evaluate the apoptosis of the myocardial tissues [16]. Firstly, heart sections were fixated with 4% *w*/*v* formaldehyde solution for 10 min at 25°C and then rinsed with PBS. Subsequently, the sections were postfixed in formaldehyde and acetic acid at 4°C (5 min) and then washed with PBS. An equilibration buffer and working strength deoxynucleotide transferase were then added successively, and the sections were maintained at 37°C for 1 h. Following washing, the sections were immersed into Hoechst 33342 at 25°C (5 min). Negative control was just added a TUNEL reaction mixture. The examination of slides was performed at ×200 magnification, and photographs were obtained with an epifluorescence microscope (Nikon Eclipse 80i). The TUNEL-positive cells are expressed as the percentage.

### 2.10. Hoechst Staining

Hoechst staining was performed following the methods of Mao et al. to evaluate the apoptosis of H9C2 cells [16]. First, the cells were fixed with formaldehyde (4%) at 25°C for 10 min. After that, the formaldehyde was removed and the cells were rinsed twice with PBS. Then, the cells were incubated with Hoechst 33258 (Beyotime Institute of Biotechnology) at 25°C for 5 min. Following that, pictures of cell apoptosis were taken using a fluorescent microscope (Olympus; magnification: ×200) and cellular apoptotic percentage was evaluated as the following formula: Number of apoptosis bodies/(number of apoptotic cells + number of cells).

### 2.11. Flow Cytometry Analysis

Flow cytometry was used for assessing the apoptosis of H9C2 cells following the methods established by Mao et al. [16] First, H9C2 cells were treated with FITC-conjugated Annexin V (C1062M, Beyotime Institute of Biotechnology, 2.5%, *v*/*v*) and propidium iodide (PI) (5%, *v*/*v*) in dark at 25°C for 20 min. Thereafter, the cellular apoptosis and death of H9C2 cells were analyzed using flow cytometry (BD FACSCalibur; BD Biosciences). Cell apoptosis was analyzed using CellQuest Pro software (BD FACSCalibur; BD Biosciences; US): Cell death percentage = the percentage of early plus late apoptotic cells.

### 2.12. Real-Time PCR

Real-time PCR analysis was performed following the methods of Mao et al. [16] Total RNAs from myocardial tissues or H9C2 cells were extracted using TRIzol (Takara Biotechnology Co., Ltd.). The purity and concentration of RNA were determined using NanoDrop One spectrophotometer (ThermoFisher). Prior to PCR, the cDNAs were obtained using a reverse transcription kit (cat. no. DRR037A; Takara Bio, Inc.). Real-time PCR was conducted with ABI 7300 plus system (Applied Biosystems; Thermo Fisher Scientific, Inc.) using SYBRTM Green PCR Kit (Takara Bio, Inc.). *β*-Actin was defined as the internal reference. The 2-*ΔΔ*Cq method was used for data analysis, and results were expressed as the ratio of NOX2 mRNA to *β*-actin mRNA or miR-532-3p to U6 [17]. The following primers were used: miR-26a-5p forward: 5′-CGGCGGTTTTTGCGGTCTGGGCT-3′, reverse: 5′-GTGCAGGGTCCGAGGT-3′; PTEN forward: 5′ -AAGACCATAACCCACCACAGC-3′, reverse: 5′-ACCAGTTCGTCCCTTTCCAG-3′; *β*-actin forward, 5′-CCCATCTATGAGGGTTACGC-3′ and reverse, 5′-TTTAATGTCACGCACGATTTC-3′.

### 2.13. Western Blotting

Western blotting analysis was performed following the methods of Mao et al. [16] Total protein from heart tissues or H9C2 cells was extracted using a cell lysis buffer (cat. no. P0013; Beyotime Institute of Biotechnology), and the protein concentration was determined using the bicinchoninic acid protein assay kit (cat. no. P0009; Beyotime Institute of Biotechnology). 40 *μ*g of protein from each sample was used for western blotting analysis. First, the protein was separated via 10% SDS-PAGE and transferred to PVDF membranes. Then, the membranes were occluded with 5% fat-free milk for 2 h and rinsed twice with PBS, each 5 min. After that, the PVDF membranes were incubated with primary antibodies (1 : 1000) against rabbit anti-PTEN (Santa Cruz Biotechnology, Inc.) or anti-caspase-3 (Santa Cruz Biotechnology, Inc.) or anti-AKT or anti-p AKT or *β*-actin (Santa Cruz Biotechnology, Inc.) for 16 h at 4°C in dark. Then, these membranes were rinsed twice with water (each 5 min) and incubated with a horseradish peroxidase-conjugated goat anti-rabbit secondary antibody (Beyotime Institute of Biotechnology; 1 : 2000) for 2 h at 25°C. Then, enhanced chemiluminescence solutions (BeyoECL Plus kit; Amersham; Cytiva) and a ChemiDox XRS+ Imaging System (Bio-Rad Laboratories, Inc.) were used for protein visualization and imaging. ImageJ 1.43 (NIH) was used for densitometric analysis of protein bands. Results were expressed as the ratio to *β*-actin.

### 2.14. Statistical Analysis

Data are reported as means ± SD and were assessed by GraphPad Prism Software (version 7, USA). Each experiment was performed repeatedly at least three times. The difference between two groups was evaluated by Student's *t*-test, and the comparison between more than two groups was analyzed by one-way ANOVA with Bonferroni's multiple comparisons test. A value of *P* = 0.05 was regarded as a significant difference.

## 3. Results

### 3.1. I/R Injury Contribute to Myocardial Infarction and Apoptosis

**Figure 1:**
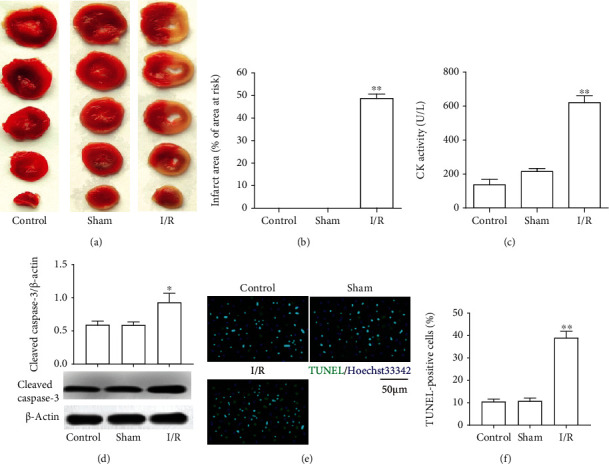
I/R injury contribute to myocardial infarction and apoptosis. (a) TTC staining. (b) Infarction area. (c) CK activity. (d) Cleaved caspase-3 protein expression. (e) TUNEL/Hoechst double staining. (f) TUNEL-positive cell counting. The differences of the values between the groups were analyzed by one-way ANOVA. All experiments were repeated 3 times, and all data are mean ± standard deviation. ^∗^*P* < 0.05 vs. the control; ^∗∗^*P* < 0.01 vs. the control.

To simulate myocardial ischemia clinically, we established a rat MIRI model. As Figures 1(a) and 1(b) show, the rats subjected to MIRI presented significant myocardial infarction (TTC staining of heart tissues is white). Then, we measured the activity of creatine kinase (CK) which is an important biomarker of acute myocardial infarction and found that rats underwent MIRI with higher CK activity when compared with control (Figure 1(c)). This suggested that MIRI caused myocardial cell death. Consistently, heart tissues subjected to MIRI with higher expression of cleaved caspase-3(Figure 1(d)). Similarly, the TUNEL/Hoechst staining indicated that I/R obviously increased the apoptosis of heart tissues when compared to the control (Figures 1(e) and 1(f)). These data above indicated that MIRI resulted in cell apoptosis.

### 3.2. The Effect of I/R on PTEN/AKT Signaling

**Figure 2:**
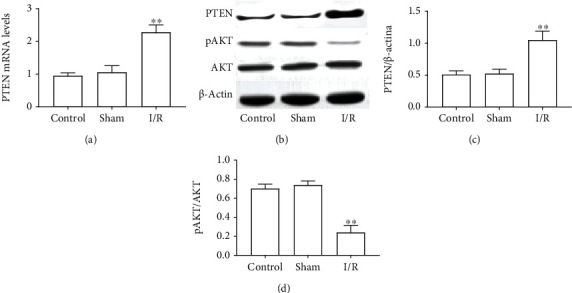
The effect of I/R on PTEN/AKT signaling. (a) mRNA level of PTEN. (b) Protein expression of PTEN, AKT, and pAKT. (c) The ratio of PTEN to *β*-actin. (d) The ratio of pAKT to AKT. The differences of the values between the groups were analyzed by one-way ANOVA. All experiments were repeated 3 times, and all data are mean ± standard deviation. ^∗∗^*P* < 0.01 vs. the control.

As AKT signaling is an important prosurvival pathway, its abnormal was closely associated with cell apoptosis. Our next experiment was designed to observe the effect of MIRI on PTEN/AKT signaling. Compared to the control group, the expression level of PTEN was significantly increased at both mRNA and protein in MIRI rats (Figures 2(a)–2(c)). Consistently, the phosphorylation of AKT in MIRI rats was significantly decreased, compared to the control group (Figures 2(b) and 2(d)). These data suggested that the high expression of PTEN contributed to insufficient of AKT phosphorylation was one of the main causes of MIRI.

### 3.3. Bioinformatics Analysis and the Effect of I/R on the Expression of miR-129

**Figure 3:**
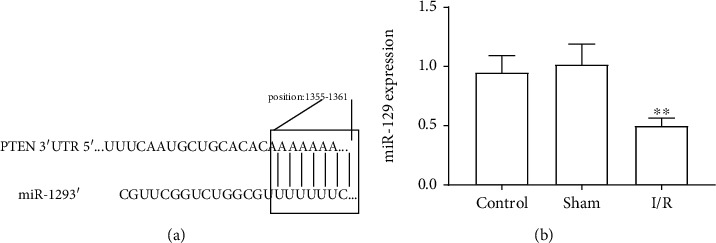
Bioinformatics analysis and the effect of I/R on the expression of miR-129. (a) Schematic diagram of bioinformatics analysis. (b). miR-129 expression level. The differences of the values between the groups were analyzed by one-way ANOVA. All experiments were repeated 3 times, and all data are mean ± standard deviation. ^∗∗^*P* < 0.01 vs. the control.

Considering that miRNAs has an important role in gene expression and is involved in many cellular functions, such as cell apoptosis, we then focused on the role of miRNA in PTEN expression regulation. Through bioinformatics analysis, we found that PTEN is a putative target of miR-129 (Figure 3(a)). As the present data show, compared to the control, the expression of miR-129 was significantly decreased in MIRI rats (Figure 3(b)). This indicated that there is a reverse relationship between the expression level of miR-129 and PTEN.

### 3.4. The Effect of miR-129 on the Relative Luciferase Activity

**Figure 4:**
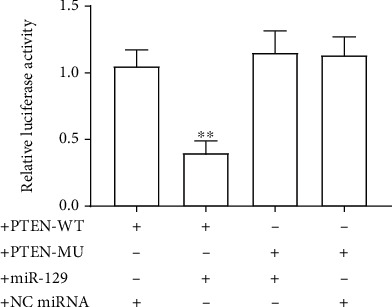
The effect of miR-129 on the relative luciferase activity. Relative luciferase activity. The differences of the values between the groups were analyzed by one-way ANOVA. All experiments were repeated 3 times, and all data are mean ± standard deviation.^∗∗^*P* < 0.01 vs. the PTEN-WT+NC miRNA.

To further confirm the relationship between miR-129 and PTEN, we then performed a dual luciferase activity experiment. As shown in Figure 4, miR-129 mimics significantly decreased the relative luciferase activity of cells transfected with wild-type plasmid PTEN-WT (containing a wild seed sequence “UAAAAAA” of PTEN 3′UTR), but not cells transfected with mutated plasmid PTEN-MU (containing a mutated seed sequence “UAAGAAA” of PTEN 3′UTR). Additionally, NC miRNA did not affect the relative luciferase activity of cells transfected with PTEN-WT or PTEN-MU plasmid. These data suggested that PTEN is a target of miR-129.

### 3.5. miR-129 Mimics Reversed the Effect of H/R on PTEN/AKT Signaling in H9C2 Cells

**Figure 5:**
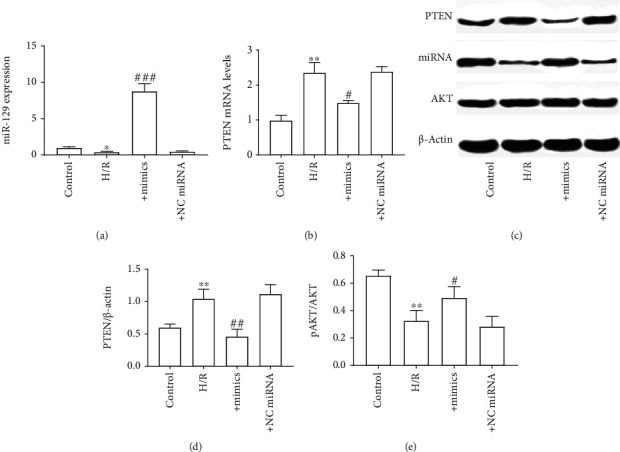
miR-129 mimics reversed the effect of H/R on PTEN/AKT signaling in H9C2 cells. (a) miR-129 expression level. (b) mRNA level of PTEN. (c) Protein expression of PTEN, AKT, and pAKT. (d) The ratio of PTEN to *β*-actin. (e) The ratio of pAKT to AKT. The differences of the values between the groups were analyzed by one-way ANOVA. All experiments were repeated 3 times, and all data are mean ± standard deviation. ^∗^*P* < 0.05 vs. the control; ^#^*P* < 0.05 vs. H/R. ^##^*P* < 0.01 vs. H/R. ^###^*P* < 0.001 vs. H/R.

To simulate MIRI in vitro, H9C2 cells were subjected to H/R according to the methods described above. Consistent with the rat MIRI model, H/R significantly decreased the expression level of miR-129 in H9C2 cells (Figure 5(a)), accompanied with an increased expression level of PTEN (Figures 5(b)–5(d)) and insufficient phosphorylation of AKT (Figures 5(c) and 5(e)). Our next experiments were designed to observe whether miR-129 mimics can reverse the effect of H/R on PTEN/AKT signaling. As shown in Figure 5(a), miR-129 mimic (not NC miRNA) transfection significantly increased the expression level of miR-129 in H9C2 cells. This suggested that miR-129 mimics were successfully transfected into cells and work properly. As expected, miR-129 mimics significantly reversed the phenomenon that H/R induced an increase of PTEN expression in H9C2 cells, as well as decrease of AKT phosphorylation (Figures 5(b)–5(d)).

### 3.6. miR-129 Mimics Reversed the Effect of H/R on Apoptosis in H9C2 Cells

**Figure 6:**
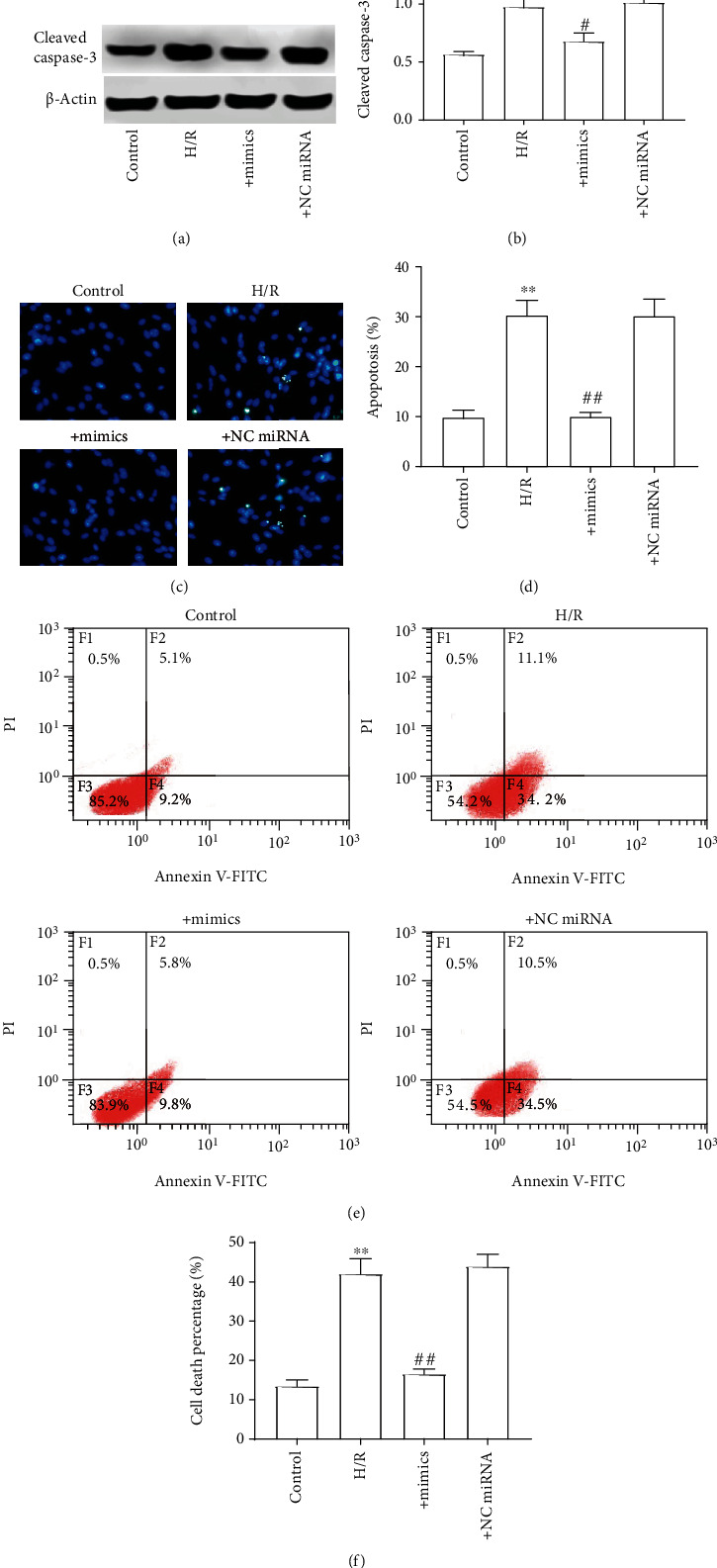
miR-129 mimics reversed the effect of H/R on apoptosis in H9C2 cells. (a) Cleaved caspase-3 protein expression. (b) The ratio of cleaved caspase-3 to *β*-actin. (c) Hoechst staining. (d) Apoptosis rate. (e) Presentative images of cell apoptosis by flow cytometer. (f) The percentage of cell death. The differences of the values between the groups were analyzed by one-way ANOVA. All experiments were repeated 3 times, and all data are mean ± standard deviation. ^∗^*P* < 0.05 vs. the control; ^∗∗^*P* < 0.01 vs. the control; ^#^*P* < 0.05 vs. H/R; and ^##^*P* < 0.01 vs. H/R.

We then observed the effect of miR-129 mimics on apoptosis of H9C2 cells. As shown in Figures 6(a) and 6(b), compared with the control, H/R significantly increased the expression level of cleaved caspase-3. Consistently, the Hoechst staining results show that miR-129 mimics significantly inhibited the H/R-induced apoptosis in H9C2 cells (Figures 6(c) and 6(d)). Similarly, as shown in Figures 6(e) and 6(f), the apoptosis of H/R-induced H9C2 cells was obviously inhibited by miR-129 mimics. These data indicated that miR-129 is an important molecule related to the apoptosis of myocardial cell or a potential drug for the myocardial infarction therapy.

## 4. Discussion

Myocardial ischemia reperfusion injury is a common pathophysiological process in clinic, and its detailed mechanism is still need to be elucidated. In this study, we established in vivo and in vitro models to simulate MIRI clinically and found that PTEN/AKT signaling abnormal was closely associated with myocardial cell apoptosis. We found that low expression of miR-129 accelerates the myocardial cell apoptosis by directly targeting 3′UTR of PTEN. This suggested that miR-129 may be an important biomarker for MIRI and a target for its treatment.

PTEN, an upstream molecule of AKT signaling, involves in the development and progress of many diseases, such as tumor, stroke, and acute myocardial infarction [14, 15, 18]. A great many of studies have found that low expression of PTEN was closely associated with tumor cell viability, proliferation, migration, invasion, and drug resistance and found that inhibition of PTEN pharmacologically promotes tumors cell apoptosis [15, 19, 20]. Different from cancer, studies related to ischemic stroke and myocardial ischemia injury have found that high expression of PTEN was closely associated with neural cell or cardiomyocyte death and that knockdown of PTEN protects the neuron or cardiomyocyte from ischemia injury [21, 22]. These data indicated that PTEN was a target for disease therapy (including cancer and stroke) or for drug development. Therefore, elucidating the detail mechanism of PTEN expression regulation becoming the most interested events in fundamental research or clinical research. In the past decades, a great many of studies were designed to investigate the role of miRNAs in PTEN expression regulation since miRNAs were found have crucial roles in gene expression. According to the report by Wei et al., in clinical specimens of multiple human cancers (breast cancer and bladder cancer), the expression of miR-130 family members correlated inversely with PTEN expression [23]. In another study carried out by Zheng et al., they found that miR-130a exerts neuroprotective effects against ischemic stroke through the PTEN/PI3K/AKT pathway [24]. Besides miR-130a, in fact, more and more miRNAs were found negatively regulating the expression of PTEN, such as miR-301, miR-26, miR-19, miR-29, miR-140, miR-142, and miR-200 [18, 19, 25–27]. However, most of these studies were performed on cancer, and few were down on myocardial ischemia reperfusion injury. To further understand the miRNAs/PTEN axis in MIRI, therefore, more investigations should be performed to observe the interaction between miRNAs and PTEN in myocardial ischemia reperfusion injury.

During the process of MIRI, the expression of many miRNAs was found altered (downregulated or upregulated) in myocyte, for example, miR-130a, miR-26, and miR-193, and these miRNAs were biomarkers for MIRI. In this study, we focused on miR-129 and found that the expression of miR-129 was significantly downregulated in rats subjected to MIRI or in H9C2 cells subjected to H/R. In fact, miR-129 was a multifunctional biomolecule involving in the development and progression of many diseases, such as tumor [20, 28]. Recent studies found that miR-129 plays crucial roles in cardiovascular and cerebrovascular diseases, especially in myocardial ischemia reperfusion injury; for example, Chen et al. have found that miR-129-5p protects against myocardial ischemia-reperfusion injury via targeting HMGB1, and Ma et al. found that miR-129-5p alleviates myocardial injury by targeting suppressor of cytokine signaling 2 after ischemia/reperfusion [29, 30]. In addition, Zou et al. found that miR-129 is involved in the process of inflammation and apoptosis in cardiomyocytes by directly targeting Smad3 [31]. These data above indicated that miR-129 is a potential biomarker for MIRI and a therapy target for myocardial infarction. Interestingly, the present study found that the expression of miR-129 (downregulated) was inversely correlated with the expression of PTEN (upregulated) in both the MIRI rat model and H/R cell model; this suggested that PTEN is a putative target of miR-129, and it was confirmed by miR-129 mimic transfection experiment and luciferase reporter gene experiment. As the results presented, miR-129 mimic significantly reversed the expression of PTEN in H/R-induced H9C2 cells, as well as the phosphorylation of AKT. Moreover, miR-129 mimic significantly decreased the relative luciferase activity. These data demonstrated that miR-129 is involved in MIRI by directly targeting PTEN. However, different from our findings, Xie et al. have found that miR-129 can inhibit the phosphorylation of AKT by binding to the 3′UTR of PI3KCA and LINC00198 as a sponge of miR-129 play roles in PTEN expression inhibition by regulating the formation of REST, RCOR1, and HDAC2 [32]. This suggested that miR-129 can regulate AKT phosphorylation from many ways. In addition, considering mTOR is the direct downstream of PTEN-Akt signaling and a key protein responsible for the regulation of many signaling pathway, such as proliferation- and apoptosis-related pathways, we predict miR-129 may be a potential target for salvage of myocardial ischemia in the future [33]. However, before miR-129 as a drug or therapy used clinically, there are a lot of works still need to be done. There are many limitations of this study; for example, the function study of miR-129 only performed in an in vitro model and whether it also come into play in vivo model are still not known. In addition, MIRI is a pathological process that was closely associated with metabolism dysfunction of the myocardium. So, whether miR-129 is involved in the mitochondrial oxidative phosphorylation process deserved further exploring.

This study firstly demonstrated that miR-129 ameliorates myocardial cell apoptosis by directly target 3′UTR of PTEN. This suggested that the miR-129/PTEN axis is a potential target for MIRI therapy.

## Data Availability

The dataset used and/or analyzed during the current study are available from the corresponding author on reasonable request.

## Supplementary Materials

Supplementary Materials Figure S1: image of plasmids electrophoresis. Representative image of plasmid electrophoresis. pGL6: vector plasmid; PTEN-WT: plasmid containing the wild-type (WT) binding sites of miR-129-5p; PTEN-MU: plasmid containing the mutant (MUT) binding sites of miR-129-5p; control: water.
