# Supplementary figures and images for: miR-129 Attenuates Myocardial Ischemia Reperfusion Injury by Regulating the Expression of PTEN in Rats
Source: Biomed Res Int. 2021 Aug 14;2021:5535788. doi: 10.1155/2021/5535788 (PMC8382530; doi:10.1155/2021/5535788)

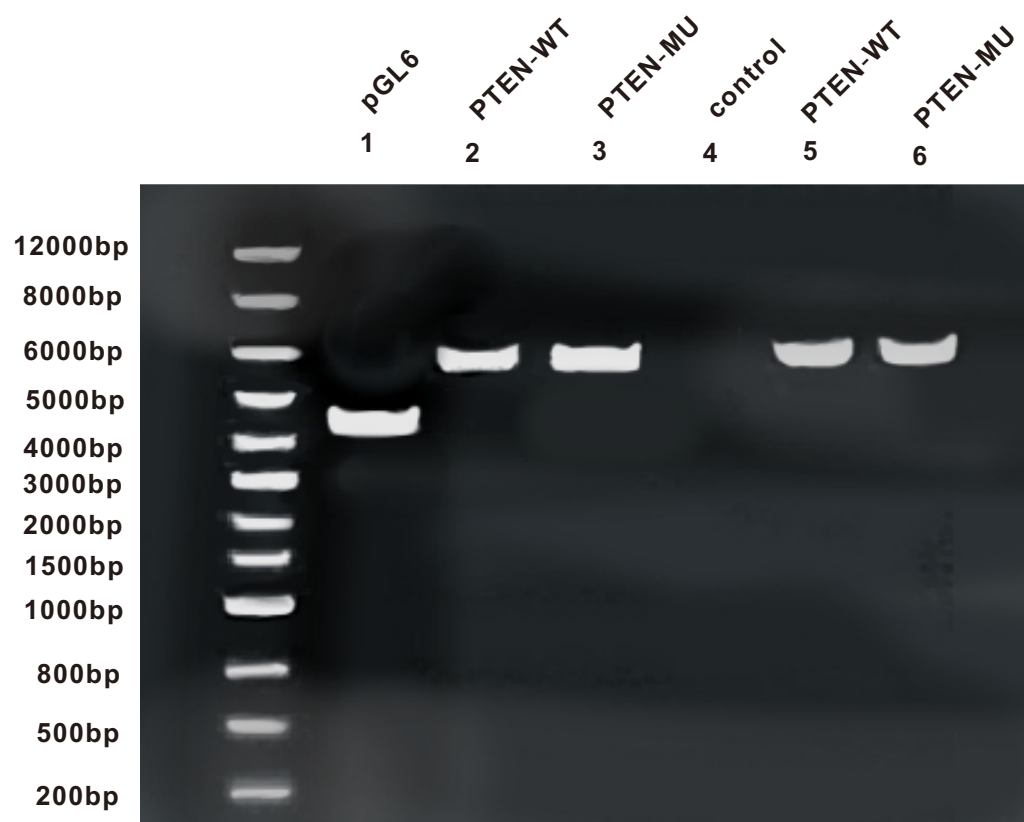

figureS

Supplement: Supplementary Materials — Figure S1: image of plasmids electrophoresis. Representative image of plasmid electrophoresis. pGL6: vector plasmid; PTEN-WT: plasmid containing the wild-type (WT) binding sites of miR-129-5p; PTEN-MU: plasmid containing the mutant (MUT) binding sites of miR-129-5p; control: water. [file 5535788.f1.pdf]
